# Living at the Wrong Time: Effects of Unmatching Official Time in Portugal and Western Spain

**DOI:** 10.3390/biology11081130

**Published:** 2022-07-28

**Authors:** María-Ángeles Bonmatí-Carrión, Elvira Casado-Ramirez, María-Teresa Moreno-Casbas, Manuel Campos, ModulEN Consortium, Juan Antonio Madrid, Maria-Angeles Rol

**Affiliations:** 1 Chronobiology Laboratory, Department of Physiology, College of Biology, University of Murcia, Mare Nostrum Campus, IUIE, IMIB-Arrixaca, 30100 Murcia, Spain; jamadrid@um.es; 2 Ciber Fragilidad y Envejecimiento Saludable (CIBERFES), 28029 Madrid, Spain; e.casado@externos.isciii.es (E.C.-R.); mmoreno@isciii.es (M.-T.M.-C.); manuelcampos@um.es (M.C.); 3 Unidad de Investigación en Cuidados y Servicios de Salud (Investén-Isciii), Instituto de Salud Carlos III, 28029 Madrid, Spain; 4 Artificial Intelligence and Knowledge Engineering Group, INTICO, University of Murcia, 30100 Murcia, Spain

**Keywords:** circadian rhythms, official time, wrist skin temperature, light exposure, sleep, social jet lag, circadian disruption

## Abstract

**Simple Summary:**

Human rhythmicity is subjected to the workings of the internal circadian clock, but it is also influenced by environmental time (mainly the light-dark cycle) and social timing imposed by the official time at our location, as well as by our work schedule. When a misalignment among these times occurs, an internal order impairment appears, which affects our health. Western Spain (GMT+1/+2) and Portugal (GMT0/+1) share similar longitudes (sun time) but have different official times, and thus they provide a “natural experiment” to assess how this discrepancy affects circadian rhythmicity and sleep in people with no work duties (>65 years). Although sleep duration was not affected, the circadian rhythms in the Portuguese were more robust, especially during weekdays, while higher desynchronization tended to occur in the Spaniards. Once official time was corrected by GMT0, meals took place later in Spain than in Portugal, especially as the day progressed, indicating the possible deleterious effect on circadian system robustness when official time is misaligned with its corresponding geographical time zone.

**Abstract:**

Human circadian rhythmicity is subjected to the internal circadian clock, the sun and social clocks (official time, social/work schedules). The discrepancy among these clocks, as occurs when official time does not match its geographical time zone, may produce circadian disruption. Western Spain (GMT+1/+2) and Portugal (GMT0/+1) share similar longitudes (sun time) but have different official times. This provides a unique opportunity to evaluate the effects of official time on circadian rhythmicity and sleep in elderly and retired populations (with no remunerated duties presumed, although other social commitments may be present) at both locations. Although both populations slept enough for their age (7–8 h), circadian robustness (e.g., interdaily stability, relative amplitude) was greater in Portugal, especially during weekdays, while greater desynchronization (both body temperature vs. motor activity and body temperature vs. light exposure) tended to occur in the Spaniards. Once corrected by GMT0, meals took place later in Spain than in Portugal, especially as the day progresses, and a possible interplay between bed/meal timings and internal desynchronization was found. Our results point to the possible deleterious effect on circadian system robustness when official time is misaligned with its geographical time zone.

## 1. Introduction

Human circadian rhythmicity is subjected to three clocks: internal (our body clock or circadian system), environmental (the sun clock, natural light-dark cycle) and social (official time and social/work schedules). Our circadian status will depend on the interplay among these three clocks or time frames [1], and thus the lack of synchronization between them could lead to social jet lag (when a discrepancy in weekday-workday sleep patterns occurs) and internal temporal misalignment or chronodisruption. This condition has been associated with a higher incidence of psychiatric, cardiovascular, metabolic and cellular disorders, including some types of cancer (reviewed in [2]), among other health concerns.

In mammals, the circadian system consists of a circadian pacemaker located in the suprachiasmatic nuclei (SCN) of the hypothalamus, which is responsible for sending rhythmic temporal signals to the different organs and peripheral clocks of tissues [3]. Under natural conditions, the central pacemaker is essentially entrained by the light-dark cycle (for a review, see [4]). This cyclic signal acts as a *zeitgeber*, synchronizing the circadian system through the retinohypothalamic tract starting at the intrinsically photosensitive retinal ganglion cells (ipRGCs). These ipRGCs contain melanopsin, a photopigment sensitive to 460–480 nm light [5,6], and also receive inputs from rods and cones (extrinsic pathway) [7,8,9,10]. Thus, the assessment of individual light exposure patterns, considering not only total, but also circadian (460–480 nm) stimuli, is of interest when evaluating circadian status in humans [11].

Apart from the evident interest in assessing how people are exposed to the main circadian inputs, the human circadian system needs to be assessed through the study of its outputs. Among them, we can mention the daily patterns of the secretion of different hormones, with melatonin rhythm being considered as the gold standard for circadian phase assessment; the sleep-wake cycle, which can be assessed directly through polysomnography or indirectly through actigraphy (recording the motor activity (MA) patterns) [12]; and skin temperature, which, although affected by masking, shows a greater endogenous component than motor activity [13,14]. In fact, skin wrist temperature has recently been proposed as a reliable indicator to be used instead of dim light melatonin onset (DLMO) to predict the internal phase [13,15] (with the proposed wrist temperature increase onset, WTiO [13]). 

The social clock reflects local (official) time, a social construct referring to the sun time at the meridian chosen for that time zone. Meridians divide the Earth into 360 “orange sections” of one degree each and serve to establish different time zones. These zones comprise around 15 degrees, spanning half an hour of solar time (7.5 longitudinal degrees) to the east of the divisor line and half an hour to the west. Time zones are all referenced to Greenwich Meridian Time (GMT), with negative hours to the west and positive to the east [1].

However, different economic and political reasons have led time zones to differ from their geographical areas in some cases. For example, China is officially in just one time zone, although the entire country would, theoretically, cover five time zones (GMT+5–GMT+9). On the contrary, the United States of America (excluding Alaska), with a similar extension, comprises four official time zones (GMT-5–GMT-8) (with Alaska being officially at GMT-9, although geographically, it should be GMT-10), while Europe presents five (GMT+3–GMT-1). However, in this case, the official time zones do not always match the geographic time zones. As an example, France and Spain should be GMT0, together with England, Ireland and Portugal.

This discrepancy between environmental and social clocks has received increasing attention from epidemiologists, who examine people living within a specific time zone. Indeed, living towards the west has been found to be a predictor of a higher incidence of cancer and mortality and shorter life expectancy at birth [16,17,18]. These epidemiologically-assessed risks have been attributed to a continuous circadian disruption, as proposed by Stevens (2005) [19]. In this sense, meal times, influenced by both internal and environmental clocks, but also by social and cultural references, have been scarcely studied in relation to circadian disruption, although their effects on metabolic health have been established [20,21,22,23,24,25,26].

Chronotype (internal clock), assessed through questionnaires [27,28], has been previously found to be associated with different external factors, such as latitude and longitude [29]. Chronotype becomes later as we move towards the west, and thus the potential social jet lag experienced within the same time zone could be greater for “westerners”. This situation becomes even more marked when time zones do not match their corresponding theoretical and geographical areas, as is the case of Spain, which is officially assigned to GMT+1/+2 (+2, due to Daylight Saving Time, DST), but is geographically located in the western half of GMT0. It can be even worse in the West of Spain, with sunrises and sunsets occurring much “later” than in Central Europe, with which it shares official time.

Variations in chronotype, potential jet lag and associated health risks have been assessed worldwide through questionnaires and epidemiological studies, respectively, finding that a later chronotype and higher social jet lag is present with both western longitudes and location within time zones [29]. Social schedules, like school or work, are also known to interfere with individual sleep preferences and circadian synchronization. Indeed, social jet lag is defined as the difference in sleep phase between working and free days [30]. Non-working populations living under different official times, but with similar solar times, as occurs in western Spain and Portugal, offer a unique opportunity to isolate (at least partially) the effect on the circadian system of this solar versus official time lag from that due to working schedules. Furthermore, and as far as we know, although actigraphy has been previously used to assess bedtime [31], to date, there are no studies based on objective multivariable recording tools to assess the circadian status and chronodisruption in people living in border areas with similar sun times and different official times. This is a matter of increasing interest due to the recent European proposal to abolish Daylight Saving Time, implying that each country must choose which standard time zone to maintain throughout the year.

Thus, our aim was to evaluate the effect of official time on objectively assessed circadian rhythmicity and inferred sleep (internal time) in retired populations living in locations with similar longitude (similar environmental time) and different official times (different social time, Portugal: GMT0/+1, and western Spain: GMT+1/+2). In addition, we evaluated the possible association between bed and meal timing in circadian synchronization.

## 2. Materials and Methods

### 2.1. Study Population

For the present study, 294 participants residing in western Spain (71.8 ± 4.8 y/o, mean ± SD; 140 women, 1 missing value) and 135 residing in Portugal (72.1 ± 4.7 y/o; 99 women, 2 missing values) were recruited. Participants were recruited by their primary care nurse when they visited their corresponding primary health center for other follow-up care. It was a convenience sample without any randomization. Those with a Bartel Index ≥ 60, or the sum of three frailty questions = 3, or who were unable to perform the necessary procedures, or those with a moderate/severe cognitive deficit were excluded. Participants were recorded with an ambulatory monitoring device for one week. Volunteers received appropriate information about the study protocol and signed a written informed consent form in accordance with the Helsinki Declaration of 1975, as revised in 2008, before being enrolled into the study. This research project was approved by the University of Murcia Ethics Committee (ID 2072/2018), and all research was performed in accordance with relevant guidelines/regulations.

### 2.2. Locations and Period of Recording

**Figure 1:**
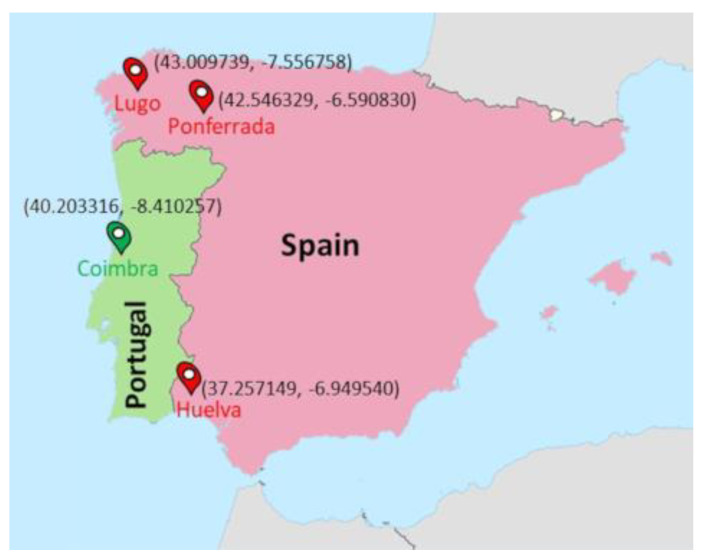
Map of the participants’ locations: Lugo (43.009739, −7.556758), Ponferrada (42.546329, −6.590830) and Huelva (37.257149, −6.949540) for Western Spain; Coimbra (40.203316, −8.410257) for Portugal. See Methods for coordinates. Modified from a file licensed under the Creative Commons Attribution-Share Alike 3.0 Unported license (author: NordNordWest, modifications by user: Sting).

Participants from the West of Spain resided in three locations: 91 participants resided in Lugo (43.009739, −7.556758), 117 participants resided in Ponferrada (42.546329, −6.590830) and 86 participants resided in Huelva (37.257149, −6.949540), while the participants from Portugal all resided in Coimbra (40.203316, −8.410257) (Figure 1). All towns were geographically located in a range of 1.82 and 5.75 degrees longitude and latitude, respectively. Coimbra (Portugal) was located approximately in the center of the latitudes of the Spanish towns. The recordings were performed from August 2018 to June 2019, with a similar distribution of participants in both Portugal and Spain (for details, see Table S1). Most of the recordings included both weekdays and weekends, considered as work and free days, respectively, and were analyzed both separately and in combination.

### 2.3. Ambulatory Circadian Monitoring Device

**Figure 2:**
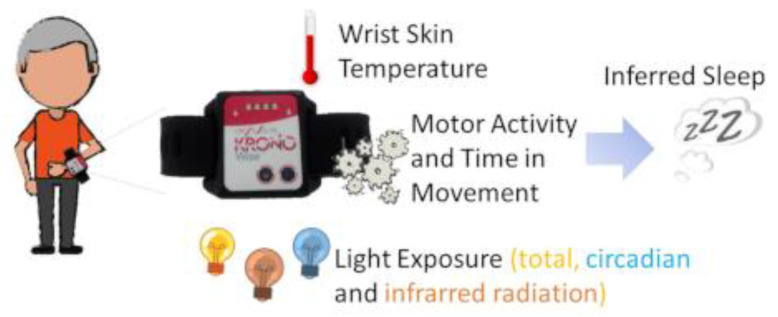
Kronowise 3.0 (ambulatory circadian monitoring system): location and variables measured. See Methods for details.

A small, watch-like device for ambulatory circadian monitoring (ACM), “Kronowise 3.0” (Kronohealth SL, Spain, Figure 2), was placed on the non-dominant hand in order to reduce masking by motor activity on circadian variables. Wrist skin temperature, triaxial motor acceleration, wrist posture and light exposure in three spectral bands (visible, blue in 460–490 nm and infrared, >800 nm) were continuously recorded at 10 (acceleration), 1 (skin temperature and light exposure), or 0.033 Hz (1 reading per epoch) for wrist position. Data for 1 week were then processed and saved into 30 s epochs. A total of 23,000,000 items of raw data were internally recorded and processed, and 230,000 of them were saved in a txt file for further analysis. For details on the ambulatory circadian device, see Supplementary Information. Participants received appropriate information about the use of Kronowise 3.0, which simply consisted of wearing it all the time (except for personal hygiene reasons), while trying not to cover the light sensors.

From the data provided by the ACM device, we selected these variables: (a) wrist skin temperature (WT) (as a variable with a high endogenous circadian component and representative of autonomic balance at the skin vessel level); (b) movement acceleration (motor activity, MA); (c) time in movement (TM), calculated as those periods of 0.1 s, in which movement on any of the three axes was detected (particularly useful to discriminate between sleep and wake states and together with (b) offers information on a circadian variable more dependent on willingness); (d) total (TL) and (e) blue (BL) and (f) infrared (IL) light/radiation exposure, to determine the intensity and timing of the main synchronizing input to the circadian system. Infrared could be used to infer the light source the participants were exposed to.

### 2.4. Automatic Detection of Sleep and Wake States

To automatically detect sleep and wake periods, we used the recently described TAPL [32], a modification of the TAP (WT-MA-body position) algorithm [33] that integrates exposure to visible light. A TAPL value of 0 would indicate deep rest, characterized by immobility, skin vasodilation and low variability of L exposure (sleep), while 1 corresponds to a wake state, light and movement. A time period was classified as sleep when the TAPL value fell beneath a pre-set threshold, previously validated by PSG [12]. All these calculations are implemented on the *Kronowizard* platform (https://kronowizard.um.es/, accessed on 29 July 2019, University of Murcia). 

### 2.5. Circadian Parameters

In order to characterize the circadian pattern of the different variables assessed, a non-parametric analysis was performed as previously described [32,34,35], obtaining day and night phase markers. In order to complete the characterization of the participant’s circadian pattern, we also calculated the interdaily stability (IS), normalized relative amplitude (RAN), circadian function index (CFI), and circadianity index, also previously described [32,34,35,36] (for details, see Supplementary Information, SI). Wrist temperature increase onset (WTiO), a circadian phase marker based on wrist skin temperature and previously validated by our group against dim light melatonin onset (DLMO) [13], was also calculated in order to serve as a circadian reference for sleep and meal timing.

### 2.6. Internal Desynchronization

To evaluate possible differences in internal desynchronization between the two countries, internal desynchronization (*DI*) indexes were also calculated for the following variables: *WT* and time in movement (*WT*/*TM*); WT and light exposure (*WT*/*TL*, *BL* and *IL*) (total, blue and infrared); and *TM* and light exposure (*TM*/*TL*, *BL* and *IL*), as previously described [37]:

$$DI\left(WT/TM\right)=\frac{\left|M5_{WT}-L5_{TM}\right|}{12}$$

$$DI\left(WT/TL\right)=\frac{\left|M5_{WT}-L5_{TL}\right|}{12}$$

$$DI\left(WT/BL\right)=\frac{\left|M5_{WT}-L5_{BL}\right|}{12}$$

$$DI\left(WT/IL\right)=\frac{\left|M5_{WT}-L5_{IL}\right|}{12}$$

$$DI\left(TM/TL\right)=\frac{\left|L5_{TM}-L5_{TL}\right|}{12}$$

$$DI\left(TM/BL\right)=\frac{\left|L5_{TM}-L5_{BL}\right|}{12}$$

$$DI\left(TM/IL\right)=\frac{\left|L5_{TM}-L5_{IL}\right|}{12}$$

### 2.7. Reported Meal and Sleep Times

Participants also completed a questionnaire for habitual bed (bed and getting up time) and meal times (breakfast, mid-morning snack, lunch and dinner) (see Supplementary Information for details). The midpoint of food intake, fasting and time ‘in bed’ were calculated as follows:

$$\mathrm{Midpoint}\;\mathrm{of}\;{}^{\prime }\mathrm{in}\;\mathrm{bed}{}^{\prime }=\mathrm{Bed}\;\mathrm{Time}+\frac{\mathrm{Bed}\;\mathrm{Time}-\mathrm{Get}\;\mathrm{up}\;\mathrm{Time}}{2}$$

$$\mathrm{Midpoint}\;\mathrm{of}\;\mathrm{food}\;\mathrm{intake}=\mathrm{Breakfast}\;\mathrm{Time}+\frac{\mathrm{Dinner}\;\mathrm{Time}-\mathrm{Breakfast}\;\mathrm{Time}}{2}$$

$$\mathrm{Midpoint}\;\mathrm{of}\;\mathrm{fast}=\mathrm{Dinner}\;\mathrm{Time}+\frac{\mathrm{Breakfast}\;\mathrm{Time}-\mathrm{Dinner}\;\mathrm{Time}}{2}$$

Time calculations were performed according to their circular nature. The midpoints for food intake, fast and ‘in bed’ were also expressed in relation to WTiO, calculated as the difference between them and WTiO [13], in order to assess the internal phase angle of entrainment. 

### 2.8. Social Jet Lag Calculation

Social jet lag was calculated as the difference between the ‘in bed’ midpoint on weekdays and weekends [38]. 

### 2.9. Local (Official) Time Correction

Local (official) time (in Spain, GMT+1 and GMT+2, before and after Daylight Saving Time (DST) change, respectively; and, in Portugal, GMT0 and GMT+1 before and after the DST change, respectively) was transformed into GMT0 in order to simulate the same official time in both countries and to avoid apparent differences related to local time expression. This correction was applied to all phase markers and times employed in the study.

### 2.10. Statistical Analyses

The normality of the data was checked using a Kolmogorov–Smirnov test. Although visual inspection of the histograms revealed a distribution close to normal, most parameters were not confirmed as normal according to this test, so a non-parametric Mann–Whitney U test was used to compare western Spain vs. Portugal, while a Wilcoxon test was used to compare weekdays vs. weekend days within each country. Spearman’s correlations were also performed between internal desynchronization indexes and sleep and meal times. The significance level at *p* < 0.05 was Bonferroni-corrected for each case, and all results were expressed as the mean ± standard error of the mean (SEM). All calculations and statistical analyses were performed using SAS version 9.4 or SPSS v20.0 (SPSS, Inc. Chicago, IL, USA). R software was also used to create violin plots.

## 3. Results

### 3.1. Patterns of Ambulatorily Monitored Circadian Variables

**Figure 3:**
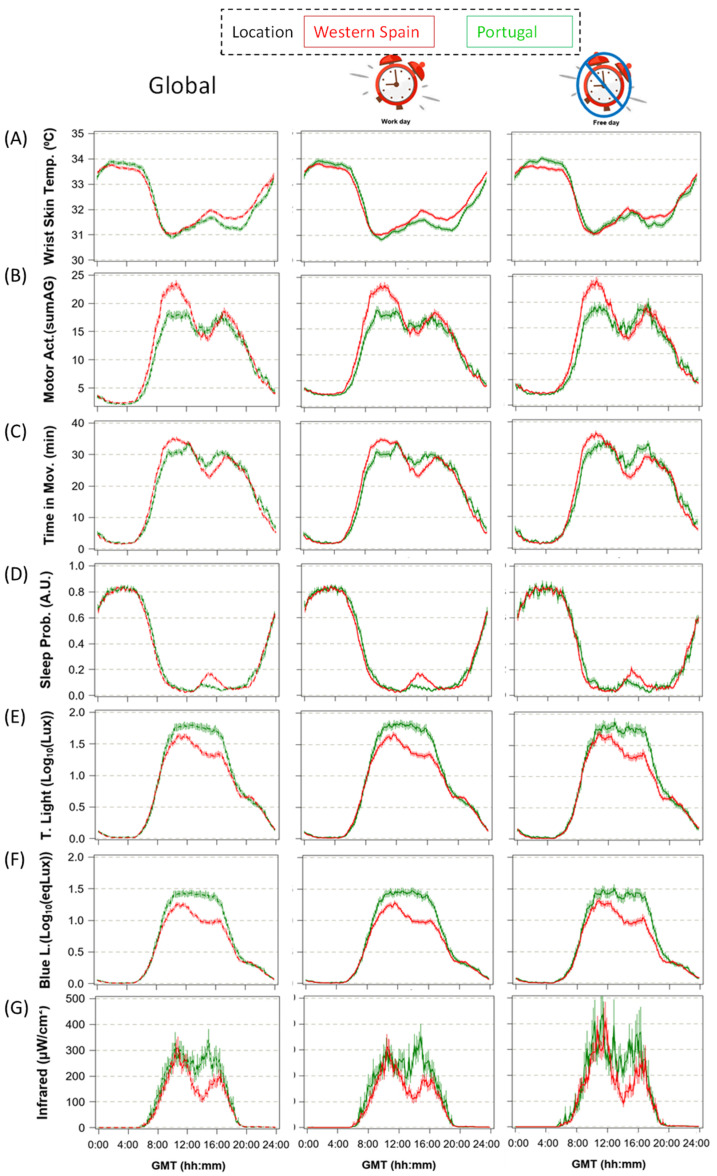
Weekly averaged mean waveforms for the whole week (left panel, 7 days), weekdays (central panel, 4–5 days) and weekend days (right panel, 2 days) in western Spanish (red, N = 294) and Portuguese participants (green, N = 135) for wrist skin temperature (**A**), motor activity (**B**), time in movement (**C**), sleep probability (**D**), total (**E**), blue (**F**) and infrared light exposure (**G**). Data are expressed as the mean ± SEM (expressed as vertical bars) and corrected by GMT0.

In order to evaluate the circadian profiles for each variable (WT, activity and light exposure), averaged mean waveforms and non-parametric indexes and phase markers, all corrected for GMT0 (Methods), were calculated for both countries (Table 1a–g). In order to assess possible differences between both day types due to social/family care commitments, all these calculations were performed for both the entire week, as well as for weekdays and weekend days separately (Figure 3). In general, most of the parameters related to stability (e.g., IS or CFI) showed better results on weekend days than on weekdays (*p* < 0.001). However, the circadianity index (CI), which indicates the proportion of circadian over ultradian components, was always higher on weekdays than on the weekend (*p* < 0.001). 

Wrist skin temperature (WT) (Figure 3A and Table 1a) showed the expected pattern, with higher values at night and lower ones during the day. When comparing curves for both countries, daytime values showed a more patent postprandial elevation (14:00–19:00) in Spanish as compared to Portuguese participants, probably related to taking an afternoon nap or “siesta”. Although this difference was partially eliminated when considering only weekend days, the normalized relative amplitude for this variable tended to be greater in the Portuguese as opposed to Spanish participants in both cases (*p* < 0.014).

Motor activity (MA, Figure 3B and Table 1b) showed a bimodal pattern with greater values in western Spain, especially during the first part of the day (10:00–12:00) (*p* < 0.004) and also at night (*p* < 0.0001), showing a tendency to greater relative amplitude (*p* < 0.015). Again, these differences partially disappeared when considering weekend days. Time in movement (TM, Figure 3C and Table 1c) showed similar profiles in both countries, with the exception of a patent postprandial decrease in western Spain, also present in sleep probability (Figure 3D and Table 1d). The amplitude for sleep probability tended to be reduced in Spain compared to Portugal (*p* < 0.045). 

Participants from Portugal were exposed to more light during the daytime (total, blue and infrared light exposure, Figure 3E–G and Table 1e–g) than the Spaniards (*p* < 0.0001), especially from midday to late afternoon (around 18:00). Therefore, total and blue light exposure also showed greater relative amplitude in Portugal than in western Spain (*p* < 0.0001).

Daytime phase markers (DPM, GMT0 corrected) were advanced in western Spain with respect to Portugal for most of the variables studied (except for sleep and infrared radiation) (Table 1). The greatest advance was observed for wrist skin temperature, while the smallest difference (albeit still significant) was found for total and blue light exposure.

Regarding the night phase markers, only WT was significantly different between the two countries when considering the weekends and the entire week, with Spanish participants showing an advance with respect to the Portuguese (*p* < 0.002). Again, WT, the most endogenous rhythm assessed, seems to be the most affected.

### 3.2. Circadian Robustness

When evaluating circadian robustness, considering interdaily stability and circadianity, participants from Portugal presented a tendency toward higher interdaily stability (IS) than those living in Spanish locations (Table 1) for sleep and all light exposure (total, blue and infrared) patterns (*p* < 0.05), while Spanish participants only tended to have greater regularities (*p* < 0.01) than the Portuguese in terms of MA. The circadianity index (CI), calculated as the ratio between the power of the first Fourier harmonic and the accumulative power of the first twelve harmonics, indicated a preponderance of the circadian over ultradian components in Portugal over western Spain in all variables measured (*p* < 0.001) except WT (Table 1a). 

Although most of the parameters followed the same tendency when comparing western Spain and Portugal, there were some differences when considering day type separately. For example, the main MA differences between the two locations disappeared (see Table 1b). This was especially patent for sleep probability and total light and infrared exposure during the weekend (Table 1e,g). In contrast, blue light exposure (Table 1f) maintained a robust difference between the two locations for both day types.

### 3.3. Circadian Desynchronization

Apart from analyzing each variable separately, the degree of desynchronization between wrist skin temperature, motor activity and light exposure, both during the entire week and also differentiating weekdays from weekend days, were also assessed.

When considering the entire week (Table 2), Spanish participants tended to present greater internal desynchronization (relative difference between each night phase marker, see Methods) between wrist skin temperature vs. time in movement and infrared exposure (0.16 ± 0.01 h) than the Portuguese (0.10 ± 0.01 h) (*p* = 0.008 and *p* = 0.014, respectively). 

Analyzing both day types separately, Spanish participants exhibited a tendency toward greater desynchronization (WT/TM, WT/TL, WT/BL, WT/IL) than the Portuguese (Table 2) (*p* < 0.03) during weekdays, while during the weekend, both groups exhibited similar levels of desynchronization. Thus, differences between the countries seemed to be specifically limited to weekdays.

### 3.4. Objectively Inferred Sleep Duration

The Spanish and Portuguese volunteers evaluated slept a similar number of hours, although participants from Portugal tended to sleep slightly longer at night (7:31 ± 0:06 h on weekend days; 7:22 ± 0:05 h on weekdays) than those from West Spain (7:25 ± 0:04 h on weekend days; 7:10 ± 0:03 h on weekdays). Interestingly, when comparing both day types, only Spaniards slept longer at night during the weekend than on weekdays (*p* < 0.05). Total sleep duration (thus including daytime sleep) was statistically longer for weekend days vs. weekdays in both western Spain (7:56 ± 0:04 h vs. 7:40 ± 0:04 h) and Portugal (7:57 ± 0:07 h vs. 7:35 ± 0:06 h) (*p* < 0.05).

### 3.5. Daily Schedule: Bed and Meal Times

**Figure 4:**
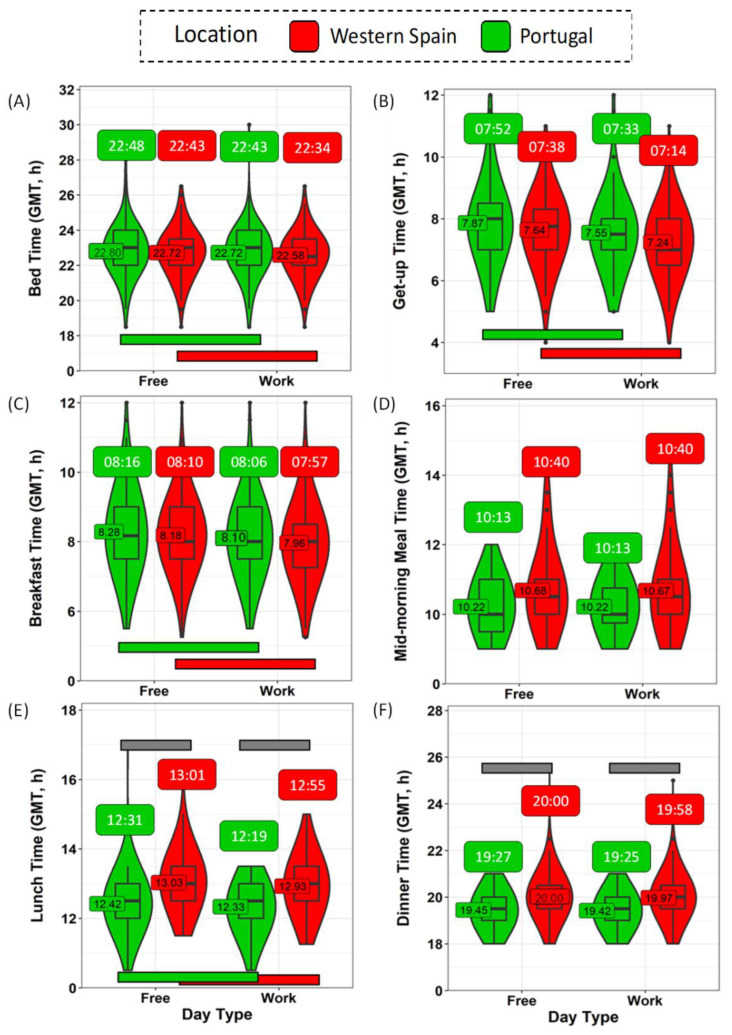
Time schedules during weekdays and weekends for participants in western Spain (red) and Portugal (green) for bedtime (**A**), getting up time (**B**), breakfast (**C**), mid-morning snack (**D**), lunch (**E**), and dinner (**F**) times. Violin plots represent kernel density estimation, with median, first and third quartiles represented in boxplots. The mean is indicated as a number. Green and red horizontal bars indicate statistically significant differences between weekends and weekdays in Portugal and western Spain, respectively. Grey horizontal bars indicate statistically significant differences between western Spain and Portugal within the same day type. Data are corrected by GMT0 and expressed in hours (both decimal and hh:mm formats).

Usual bed and meal times, examined separately for weekdays and weekends, were collected through a questionnaire. It should be noted that all data, originally expressed as local (official) time on the questionnaires, were converted into GMT0 in order to simulate the same official time for both countries (Figure 4 and Table S2). 

Bedtime occurred (Figure 4A and Table S2), in general, later on the weekends than on the weekdays, in both western Spain (22:43 ± 0:04 h vs. 22:34 ± 0:04 h, *p* < 0.001) and Portugal (22:48 ± 0:07 h vs. 22:43 ± 0:07 h, *p* = 0.008), with no differences between the countries (*p* > 0.05). In general, both populations got up (Figure 4B and Table S2) earlier on weekdays than on the weekend (*p* < 0.001). In this case, considering the GMT0 correction, Spanish participants tended to get up earlier on weekdays than the Portuguese (7:12 ± 0:04 h vs. 7:33 ± 0:06 h, *p* = 0.02). Except for the mid-morning snack and dinner, the remaining meals (Figure 4C–F and Table S2) occurred significantly earlier on weekdays than during the weekend (*p* < 0.008). 

**Figure 5:**
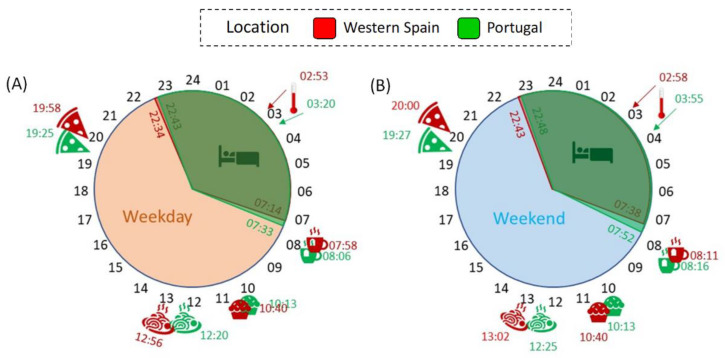
Summary of the daily schedule in Portugal (green) and western Spain (red) on weekdays (**A**) and weekend (**B**) days. A bed represents the time spent in bed; a cup of tea indicates breakfast time; a muffin represents the mid-morning snack; a pasta dish shows lunchtime; a pizza slice indicates dinner time; and a thermometer indicates the night phase marker (time for WT M5) for wrist skin temperature.

When comparing the two countries (Table S2), meal times progressively diverged throughout the day. Therefore, at the beginning of the day, although Spanish participants showed a slight tendency to have earlier breakfast and mid-morning snack times (Figure 4C,D), the differences between the two countries were not statistically significant. However, Spanish participants had lunch (Figure 4E) significantly later (12:55 ± 0:02 h on weekdays; 13:01 ± 0:02 h on weekends) than their Portuguese counterparts (12:19 ± 0:03 h on weekdays; 12:25 ± 0:03 h on weekends) (*p* < 0.001), which was also true of dinner (*p* < 0.001) (Figure 4F). Figure 5 shows a summary of the daily schedule in Portugal and western Spain on weekdays (A) and weekends (B).

In order to evaluate the phase of these schedules, we calculated the midpoint of ‘in bed’, food intake and fasting (Table S2), as well as these points in relation to WTiO (for details, see Methods section). The midpoint of the time in bed was no different between the two populations in absolute terms (GMT0). When considering the difference from WTiO (which makes reference to internal time), Spanish participants tended to sleep later than Portuguese, both during the week and on weekend days (*p* < 0.030). Less time elapsed between the midpoint of food intake and the skin temperature increase in Spain, while the opposite occurred for mid fast as compared to Portugal (Table S2, *p* < 0.001), both over the weekend and on weekdays. Thus, eating occurred closer to sleeping time.

### 3.6. Influence of Sleep and Meal Schedules on Circadian Desynchronization

In order to assess the possible relationship between internal desynchronization and sleep and meal schedules, correlations with the different internal desynchronization indexes (DI) (Table 3) were performed. Markers/schedules were significant and positively correlated with those desynchronization indexes only when calculated relative to WTiO (the later the meals or sleep occur in relation to the individual’s internal phase, the higher the internal desynchronization was). Surprisingly, the relative mid-intake correlation with internal desynchronization was stronger than that relative to the in-bed midpoint. 

### 3.7. Social Jet Lag

In general, social jet lag was not evident in this population, with values of 0.21 ± 0.04 h in Portugal and 0.27 ± 0.03 h in western Spain and a difference of around 4 min between the two locations (*p* = 0.043).

## 4. Discussion

To the best of our knowledge, this is the first time that multivariable objective circadian ambulatory monitoring has been performed on inhabitants of locations with similar longitudes and different official times. Our results show a compensation in most of the phase markers when the local (official) time was transformed into GMT0, with even later parameters found in the Portuguese than in the Spanish, probably due to the small variation in longitude. However, participants from Portugal showed better circadian robustness indexes than those from western Spain during the weekdays, indicating a possible conflict between social, internal and solar time. Meal times, however, occurred significantly later in Spain, even after GMT correction, a delay that becomes more patent as the day progresses. The correction to GMT0 reveals real differences in synchronization between the two populations under both official times, which is also evident when the individual’s phase is considered (based on WTiO, a phase marker based on wrist skin temperature and equivalent to DLMO [13]), highlighting the importance of the relative synchronization between different rhythms.

Variations in chronotype and potential social jet lag [29], as well as their associated health risks [16,17,39], have been previously assessed by means of questionnaires and epidemiological studies, respectively, finding a correlation between several health concerns and longitude and position within time zones. Thus, the chronotype becomes later as people live more toward the West [29]. In this sense, the mismatch between environmental and social clocks has led epidemiologists to pay attention to the position where people live within a time zone. However, our results, once corrected by GMT0, do not show later phase markers in western Spain (the western part of the time zone); rather, they show quite the opposite when compared to Portugal. 

Living towards the west within a time zone has been proposed as a predictor of higher cancer incidence or mortality and shorter life expectancy at birth [16,17,18]. These findings have been explained in the framework of the circadian disruption hypothesis proposed by Stevens (2005) [19], attributing these epidemiologically-assessed risks to the continuous challenge received by our physiological processes due to the discrepancy between environmental, social and internal clocks. However, although objective tools have been previously used to infer bedtime [31], as far as we know, this is the first time that such circadian disruption has been objectively assessed through ambulatory circadian monitoring (ACM) involving multivariable recording. This approach makes it possible to assess the internal synchronization of rhythms and then evaluate whether the temporal order is maintained [39]. It permitted us to demonstrate that, although both populations showed a similar phase according to solar time, robustness and synchronization (although the latter only showed a tendency) seemed to be compromised, probably due to the conflict between solar and social clocks.

When considering circadian robustness through circadian parameters such as amplitude, stability and variability, participants from Portugal showed, in general, better values than participants living in the West of Spain (with the exception of actigraphic variables). Although confounding factors could not be discarded, this advantage of the Portuguese was especially evident on weekdays (when they had more social commitments), thus indicating that the discrepancy between social vs. environmental and internal clocks could be responsible for the reduction in robustness in the Spaniards. Our study population was selected considering that this age group was mainly retired, and thus, no work duties were presumed. However, the number of grandparents raising or taking care of grandchildren has increased in all socioeconomic strata in recent years (reviewed in [40]). Thus, it is plausible that participants had social commitments during the weekdays that were presumably absent over the weekend.

Our results also show greater light exposure during the late afternoon in Portugal than in western Spain, which, together with infrared exposure, could indicate that the Portuguese, in general, are exposed to natural light to a greater extent than the Spanish, with a fall at lunchtime, which occurs earlier in Portugal. Although the ultimate reason for this difference in light exposure is difficult to address, and cultural aspects cannot be discarded, the earlier lunchtime in Portugal, even when GMT-corrected, could be a factor influencing this difference.

The integration of multiple variables, as previously described, allows sleep probability to be inferred for the entire week under normal living conditions without the burden of polysomnographic montages [12]. Sleep probability showed clear evidence of midday sleep (naps) that was more patent in Spanish than in Portuguese volunteers. On average, participants from both countries slept enough for their age (around 7 h during the night and between 7.5 and 8 h per day) [41]. However, only participants in western Spain showed significantly longer durations of nocturnal sleep on the weekend than on weekdays, which could once again be related to social constraints that limit nocturnal sleep during the week, especially for participants from Spain. A tendency to sleep longer could indicate compensation for a REM-sleep debt. REM sleep is more prevalent during the last third of the night [42], and thus it is susceptible to being reduced on weekdays when waking up is forced.

Meal times, influenced by internal and environmental clocks, but also by social and cultural references, have scarcely been studied in relation to circadian disruption. However, different studies have already pointed to an association between late meal timing and social jet lag [43] or changes in the daily profile of cortisol and wrist skin temperature [44]. In addition, recent studies show a relationship between late meal timing and metabolic impairments, mostly related to glucose tolerance [44,45,46]. To complete this triangle, the relationship between circadian disruption and metabolic disorders has also been clearly established [20,21,22,23,24,25,26]. In our study, this association was revealed when meal timing (mid-point of food intake or each meal timing) was considered in relation to the internal phase, such as the difference from WTiO [13]. Accordingly, a higher dissociation between body temperature rhythm and food intake schedules (late) is present in Spain. Thus, although confounding factors related to diet and other specific characteristics cannot be ruled out, our results could indicate that social constraints are responsible, including the “unmatched” official time in Spain. Furthermore, a longer nocturnal fast duration (as occurs in Portugal) could indicate a better metabolic response due to the lack of concurrence of food intake and melatonin [45,47].

This study presents certain limitations. First, we only monitored elderly participants who had retired from remunerated work obligations. Although our results may not be generalizable to other age groups, this made it possible (at least in part) to isolate the effect of the different official times by minimizing the effect of the diverse work schedules. However, as stated above, the elderly usually play an important role in family care, with social commitments related to their relatives’ work schedules on weekdays, and thus work schedules and the social duties linked to them seem not to have been completely eliminated. Therefore, it could be hypothesized that the differences in circadian-related parameters and desynchronization indexes might even be greater in the working-age population. Secondly, Spanish participants are distributed throughout three locations in the West of Spain, two in the north (Lugo and Ponferrada) and one in the south (Huelva), while participants from Portugal were only recorded in Coimbra. The differences in sun time between them are, nevertheless, markedly shorter than those due to official time. Thirdly, the gender distribution was different in the two countries, which could have affected the results. However, we actually performed analyses by sex, with no significant differences in circadian rhythmicity. Additionally, under ambulatory conditions, it is impossible to control factors like hobbies or entertainments, or the type of diet, only to mention some specific factors that are normally registered by questionnaires. Finally, phase markers and daily averaged patterns have been calculated based on GMT0. Although a sun time correction would have provided an alternative correction to perform a precise picture of the synchronizing effect of sunlight, in our case, we aimed to highlight the importance of using the same official time when comparing circadian phases. Additionally, circadian desynchronization and other robustness-related parameters, as well as differences between meal/bed timing relative to internal phase, would not be affected by sun time since they have been calculated based on the individual’s markers. 

## 5. Conclusions

In general, although sleep duration was sufficient for this age group in both countries and phase markers were compensated when the GMT0 correction was made, we found better circadian robustness parameters (and a tendency toward better synchronization) in participants monitored in Portugal (GMT0/+1) than in the west of Spain (GMT+1/+2), mainly on weekdays, generally reflecting a possible deleterious effect of the Spanish official time. Meal timing occurred later in Spain, especially as the day progressed, and we highlighted its association (probably bidirectional) with circadian desynchronization, although other confounding and cultural factors cannot be discarded. Our results also indicate the importance of considering both weekdays (work) and weekend days (free) separately when evaluating circadian aspects related to social clocks and, more specifically, sleep, since social commitments, more prevalent during weekdays and driven by official time (among other factors), may affect internal synchronization differently in the two day types. We hypothesize that working-age populations would suffer even greater internal desynchronization since their work schedules (fixed on the basis of official time) could force their daily activities to occur at the wrong “physiological” time. In order to confirm our results, further objective studies on different age groups with different work schedules and at different latitudes are needed. 

## 6. Patents

Kronowise 3.0 corresponds to the commercial version of the patent P201031894 (BOPI 22/3/2013), owned by the University of Murcia and licensed to Kronohealth, S.L.

## Figures and Tables

**Table 1:** a–g. Circadian parameters for whole-week recordings, weekdays and weekend days for wrist skin temperature (a), motor activity (b), time in movement (c), sleep probability (d) and total (e), circadian (f) and infrared (g) exposure.

**a**												
	**Wrist Skin Temperature**											
	**Whole week**				**Weekdays**				**Weekend**			
	**Western Spain**	**Portugal**	**Z**	**Pr**	**Western Spain**	**Portugal**	**Z**	**Pr**	**Western Spain**	**Portugal**	**Z**	**Pr**
DPM	12:53 ± 0:08	14:07 ± 0:12	4.57	**<0.0001**	12:47 ± 0:09	13:51 ± 0:12	3.77	**0.000**	13:22 ± 0:09	14:25 ± 0:15	3.07	**0.002**
NPM	2:45 ± 0:10	3:25 ± 0:10	3.88	**0.000**	2:52 ± 0:10	3:20 ± 0:11	2.58	0.010	2:58 ± 0:10	3:54 ± 0:15	3.10	**0.002**
V-day	31.33 ± 0.04	31.18 ± 0.06	−2.06	0.040	31.29 ± 0.04	31.10 ± 0.06	−2.40	0.017	31.35 ± 0.04	31.28 ± 0.07	−1.14	0.257
V-night	33.88 ± 0.04	33.99 ± 0.07	1.58	0.115	33.92 ± 0.05	34.04 ± 0.07	1.68	0.094	34.01 ± 0.05	34.22 ± 0.06	2.67	0.008
RAN	0.51 ± 0.01	0.56 ± 0.02	2.47	0.014	0.53 ± 0.01	0.59 ± 0.02	2.87	0.004	0.53 ± 0.01	0.59 ± 0.02	2.59	0.010
IS	0.56 ± 0.01	0.57 ± 0.02	0.69	0.494	0.61 ± 0.01	0.63 ± 0.02	1.36	0.176	0.75 ± 0.01	0.75 ± 0.01	0.39	0.701
CFI	0.65 ± 0.01	0.67 ± 0.01	1.52	0.129	0.67 ± 0.01	0.69 ± 0.01	2.19	0.030	0.72 ± 0.00	0.73 ± 0.01	1.64	0.102
CI	0.59 ± 0.01	0.60 ± 0.02	0.57	0.568	0.56 ± 0.01	0.57 ± 0.02	0.28	0.781	0.48 ± 0.01	0.51 ± 0.02	1.34	0.182
**b**												
	**Motor Activity**											
	**Whole week**				**Weekdays**				**Weekend**			
	**Western Spain**	**Portugal**	**Z**	**Pr**	**Western Spain**	**Portugal**	**Z**	**Pr**	**Western Spain**	**Portugal**	**Z**	**Pr**
DPM	13:10 ± 0:04	13:39 ± 0:06	3.30	**0.001**	13:02 ± 0:04	13:26 ± 0:07	2.70	0.007	13:27 ± 0:04	14:07 ± 0:09	3.58	**<0.0001**
NPM	3:03 ± 0:04	3:12 ± 0:07	1.10	0.270	2:57 ± 0:05	3:07 ± 0:07	1.31	0.190	3:18 ± 0:05	3:18 ± 0:09	−0.28	0.779
V-day	19.39 ± 0.36	17.27 ± 0.45	−2.95	0.003	19.60 ± 0.36	17.54 ± 0.46	−2.94	0.004	19.43 ± 0.41	17.71 ± 0.48	−1.72	0.086
V-night	2.26 ± 0.04	1.98 ± 0.05	−4.48	**<0.0001**	2.22 ± 0.04	1.93 ± 0.05	−4.56	**<0.0001**	2.11 ± 0.04	1.84 ± 0.04	−3.75	**<0.0001**
RAN	0.49 ± 0.01	0.44 ± 0.01	−2.55	0.011	0.49 ± 0.01	0.45 ± 0.01	−2.45	0.015	0.49 ± 0.01	0.45 ± 0.01	−1.33	0.183
IS	0.39 ± 0.00	0.37 ± 0.01	−2.64	0.009	0.45 ± 0.01	0.44 ± 0.01	−1.13	0.258	0.65 ± 0.01	0.64 ± 0.01	−1.94	0.054
CFI	0.67 ± 0.00	0.65 ± 0.01	−2.59	0.010	0.69 ± 0.00	0.68 ± 0.01	−1.92	0.056	0.76 ± 0.00	0.75 ± 0.01	−1.39	0.166
CI	0.59 ± 0.01	0.69 ± 0.01	6.74	**<0.0001**	0.56 ± 0.01	0.64 ± 0.01	5.24	**<0.0001**	0.49 ± 0.01	0.59 ± 0.01	6.07	**<0.0001**
**c**												
	**Time in Movement**											
	**Whole week**				**Weekdays**				**Weekend**			
	**Western Spain**	**Portugal**	**Z**	**Pr**	**Western Spain**	**Portugal**	**Z**	**Pr**	**Western Spain**	**Portugal**	**Z**	**Pr**
DPM	13:16 ± 0:04	13:51 ± 0:07	3.91	**<0.0001**	13:10 ± 0:04	13:43 ± 0:07	3.50	**<0.0001**	13:33 ± 0:05	14:18 ± 0:09	3.82	**<0.0001**
NPM	3:06 ± 0:04	3:11 ± 0:07	0.91	0.365	2:54 ± 0:04	3:04 ± 0:07	0.87	0.387	3:14 ± 0:05	3:18 ± 0:08	0.28	0.779
V-day	30.72 ± 0.35	30.65 ± 0.58	0.23	0.820	30.95 ± 0.35	30.89 ± 0.57	0.15	0.885	31.03 ± 0.39	31.54 ± 0.63	0.84	0.402
V-night	1.48 ± 0.05	1.51 ± 0.09	−0.46	0.647	1.43 ± 0.05	1.39 ± 0.08	−0.51	0.613	1.22 ± 0.04	1.22 ± 0.07	−0.22	0.825
RAN	0.73 ± 0.01	0.73 ± 0.01	0.15	0.881	0.74 ± 0.01	0.73 ± 0.01	0.28	0.783	0.74 ± 0.01	0.75 ± 0.02	0.80	0.427
IS	0.50 ± 0.01	0.48 ± 0.01	−1.55	0.123	0.55 ± 0.01	0.55 ± 0.01	−0.44	0.658	0.72 ± 0.01	0.70 ± 0.01	−2.13	0.034
CFI	0.76 ± 0.00	0.75 ± 0.01	−1.14	0.255	0.78 ± 0.00	0.78 ± 0.01	−0.51	0.611	0.84 ± 0.00	0.83 ± 0.00	−1.59	0.113
CI	0.66 ± 0.01	0.75 ± 0.01	6.38	**<0.0001**	0.64 ± 0.01	0.71 ± 0.01	5.32	**<0.0001**	0.58 ± 0.01	0.66 ± 0.01	5.26	**<0.0001**
**d**												
	**Sleep Probability**											
	**Whole Week**				**Weekdays**				**Weekend**			
	**Western Spain**	**Portugal**	**Z**	**Pr**	**Western Spain**	**Portugal**	**Z**	**Pr**	**Western Spain**	**Portugal**	**Z**	**Pr**
DPM	14:16 ± 0:06	14:25 ± 0:09	0.95	0.344	13:58 ± 0:07	14:14 ± 0:09	1.45	0.147	13:52 ± 0:07	14:16 ± 0:10	2.53	0.012
NPM	3:07 ± 0:04	3:18 ± 0:07	1.08	0.281	3:01 ± 0:05	3:11 ± 0:07	0.79	0.428	3:14 ± 0:05	3:22 ± 0:09	0.61	0.542
V-day	0.06 ± 0.00	0.04 ± 0.01	−4.40	**<0.0001**	0.06 ± 0.00	0.04 ± 0.00	**−4.12**	**<0.0001**	0.06 ± 0.00	0.05 ± 0.01	−3.13	**0.002**
V-night	0.84 ± 0.00	0.86 ± 0.01	1.69	0.091	0.85 ± 0.01	0.86 ± 0.01	**2.05**	0.041	0.86 ± 0.00	0.87 ± 0.01	0.91	0.365
RAN	0.78 ± 0.01	0.81 ± 0.01	3.41	**0.001**	0.79 ± 0.01	0.82 ± 0.01	**4.03**	**<0.0001**	0.80 ± 0.01	0.82 ± 0.01	2.02	0.045
IS	0.61 ± 0.01	0.62 ± 0.01	2.02	0.044	0.65 ± 0.01	0.67 ± 0.01	2.44	0.015	0.77 ± 0.01	0.77 ± 0.01	0.36	0.723
CFI	0.74 ± 0.00	0.77 ± 0.01	3.88	0.000	0.76 ± 0.00	0.79 ± 0.01	4.09	<.0001	0.80 ± 0.00	0.82 ± 0.01	2.58	0.010
CI	0.70 ± 0.01	0.76 ± 0.01	5.02	**<0.0001**	0.68 ± 0.01	0.74 ± 0.01	**5.29**	**<0.0001**	0.60 ± 0.01	0.63 ± 0.01	1.42	0.157
**e**												
	**Total Light Exposure**											
	**Whole Week**				**Weekdays**				**Weekend**			
	**Western Spain**	**Portugal**	**Z**	**Pr**	**Western Spain**	**Portugal**	**Z**	**Pr**	**Western Spain**	**Portugal**	**Z**	**Pr**
DPM	13:14 ± 0:03	13:27 ± 0:05	2.76	0.006	13:10 ± 0:04	13:24 ± 0:06	2.63	0.009	13:24 ± 0:04	13:46 ± 0:06	3.25	**0.001**
NPM	2:42 ± 0:04	2:51 ± 0:07	0.71	0.480	2:28 ± 0:04	2:37 ± 0:07	0.45	0.656	2:32 ± 0:05	2:39 ± 0:08	0.60	0.548
V-day	1.41 ± 0.03	1.64 ± 0.04	4.09	**<0.0001**	1.42 ± 0.03	1.66 ± 0.04	4.27	**<0.0001**	1.42 ± 0.03	1.66 ± 0.05	3.71	**<0.0001**
V-night	0.01 ± 0.00	0.01 ± 0.00	−0.09	0.932	0.01 ± 0.00	0.01 ± 0.00	−0.74	0.458	0.01 ± 0.00	0.01 ± 0.00	0.32	0.751
RAN	0.47 ± 0.01	0.54 ± 0.01	3.99	**<0.0001**	0.47 ± 0.01	0.55 ± 0.01	4.20	**<0.0001**	0.47 ± 0.01	0.55 ± 0.02	3.65	**<0.0001**
IS	0.55 ± 0.01	0.60 ± 0.01	3.17	**0.002**	0.60 ± 0.01	0.65 ± 0.01	3.55	**<0.0001**	0.75 ± 0.01	0.76 ± 0.01	1.50	0.134
CFI	0.83 ± 0.00	0.84 ± 0.00	3.08	**0.002**	0.85 ± 0.00	0.86 ± 0.00	3.62	**<0.0001**	0.89 ± 0.00	0.90 ± 0.00	1.82	0.070
CI	0.72 ± 0.01	0.79 ± 0.01	4.75	**<0.0001**	0.70 ± 0.01	0.76 ± 0.01	4.22	**<0.0001**	0.63 ± 0.01	0.71 ± 0.01	4.58	**<0.0001**
** f**												
	**Blue Light Exposure**											
	**Whole week**				**Weekdays**				**Weekend**			
	**Western spain**	**Portugal**	**Z**	**Pr**	**Western Spain**	**Portugal**	**Z**	**Pr**	**Western Spain**	**Portugal**	**Z**	**Pr**
DPM	13:08 ± 0:03	13:21 ± 0:05	2.46	0.014	13:06 ± 0:03	13:17 ± 0:05	1.90	0.058	13:17 ± 0:04	13:41 ± 0:06	3.40	**0.001**
NPM	2:36 ± 0:04	2:46 ± 0:07	0.76	0.448	2:19 ± 0:05	2:37 ± 0:08	1.36	0.176	2:27 ± 0:06	2:30 ± 0:09	0.17	0.862
V-day	1.05 ± 0.03	1.30 ± 0.04	4.92	**<0.0001**	1.05 ± 0.03	1.31 ± 0.04	5.04	**<0.0001**	1.06 ± 0.03	1.32 ± 0.05	4.24	**<0.0001**
V-night	0.00 ± 0.00	0.01 ± 0.00	0.31	0.754	0.00 ± 0.00	0.01 ± 0.00	1.02	0.307	0.00 ± 0.00	0.00 ± 0.00	−0.25	0.800
RAN	0.35 ± 0.01	0.43 ± 0.01	4.92	**<0.0001**	0.35 ± 0.01	0.44 ± 0.01	4.99	**<0.0001**	0.35 ± 0.01	0.44 ± 0.02	4.24	**<0.0001**
IS	0.51 ± 0.01	0.56 ± 0.01	4.25	**<0.0001**	0.56 ± 0.01	0.62 ± 0.01	4.40	**<0.0001**	0.72 ± 0.01	0.74 ± 0.01	1.99	0.047
CFI	0.81 ± 0.00	0.83 ± 0.00	4.28	**<0.0001**	0.83 ± 0.00	0.85 ± 0.00	4.78	**<0.0001**	0.88 ± 0.00	0.89 ± 0.00	2.38	0.018
CI	0.70 ± 0.01	0.77 ± 0.01	4.97	**<0.0001**	0.67 ± 0.01	0.74 ± 0.01	4.41	**<0.0001**	0.60 ± 0.01	0.68 ± 0.01	4.86	**<0.0001**
** g**												
	**Infrared Exposure**											
	**Whole Week**				**Weekdays**				**Weekend**			
	**Western Spain**	**Portugal**	**Z**	**Pr**	**Western Spain**	**Portugal**	**Z**	**Pr**	**Western Spain**	**Portugal**	**Z**	**Pr**
DPM	13:03 ± 0:03	13:10 ± 0:05	1.47	0.144	13:01 ± 0:03	13:09 ± 0:06	1.65	0.099	13:17 ± 0:04	13:30 ± 0:04	2.71	0.007
NPM	2:42 ± 0:05	2:53 ± 0:07	0.94	0.349	2:30 ± 0:05	2:43 ± 0:08	0.64	0.526	2:36 ± 0:05	2:37 ± 0:09	−0.13	0.899
V-day	186.00 ± 12.00	242.00 ± 20.00	3.59	**<0.0001**	179.00 ± 12.00	238.00 ± 21.00	3.63	**<0.0001**	214.00 ± 17.00	267.00 ± 26.00	3.03	0.003
V-night	0.06 ± 0.01	0.05 ± 0.01	−0.32	0.752	0.06 ± 0.01	0.05 ± 0.01	−0.51	0.608	0.04 ± 0.01	0.03 ± 0.01	0.17	0.868
RAN	1.00 ± 0.00	1.00 ± 0.00	0.67	0.502	1.00 ± 0.00	1.00 ± 0.00	1.17	0.242	0.99 ± 0.00	1.00 ± 0.00	0.91	0.360
IS	0.20 ± 0.00	0.21 ± 0.01	2.38	0.018	0.28 ± 0.01	0.30 ± 0.01	1.77	0.077	0.54 ± 0.01	0.56 ± 0.01	0.95	0.341
CFI	0.63 ± 0.00	0.64 ± 0.00	2.00	0.046	0.66 ± 0.00	0.67 ± 0.00	2.35	0.019	0.74 ± 0.00	0.76 ± 0.01	1.47	0.142
CI	0.38 ± 0.01	0.45 ± 0.01	5.12	**<0.0001**	0.35 ± 0.01	0.41 ± 0.01	4.20	**<0.0001**	0.31 ± 0.01	0.34 ± 0.01	2.88	0.004

DPM (h): Day Phase Marker = M10: the mid-point timing for the 10 consecutive hours with the highest values for motor activity, time in movement, TAPL and light exposure; L10: the mid-point timing for the 10 consecutive hours with the lowest values for wrist skin temperature and sleep; NPM (h): night phase marker; M5: the mid-point time of the 5 consecutive hours with the highest values for wrist skin temperature and sleep; L5: the mid-point time of the 5 consecutive hours with the lowest values for motor activity, time in movement, TAPL and light exposure; V-day = M10 mean value for motor activity (g/30 s), time in movement (s/30 s), TAPL (arbitrary units, A.U.) and light exposure (log_10_lux) and L10 mean value for wrist skin temperature and sleep; V-night = M5 mean values for wrist skin temperature and sleep; L5 mean value for motor activity, time in movement, TAPL and light exposure; RA: relative amplitude (A.U.); IS: interdaily stability (A.U.); CFI: Circadian Function Index (A.U.); CI: circadianity index (A.U.). Statistics: Mann–Whitney U test. Significance level: Pr < 0.002 (Bonferroni corrected). See Methods and Supplementary Information for further details.

**Table 2:** Internal desynchronization indexes for the entire week, weekdays and weekend days.

	Internal Desynchronization											
	Whole Week				Weekdays				Weekend			
	Western Spain	Portugal	Z	Pr	Western Spain	Portugal	Z	Pr	Western Spain	Portugal	Z	Pr
DI WT/TM	0.16 ± 0.01	0.10 ± 0.01	−2.67	0.008	0.16 ± 0.01	0.10 ± 0.01	−2.21	0.028	0.18 ± 0.01	0.17 ± 0.02	−0.64	0.524
DI WT/TL	0.16 ± 0.01	0.11 ± 0.01	−1.67	0.097	0.17 ± 0.01	0.11 ± 0.01	−2.51	0.013	0.18 ± 0.01	0.18 ± 0.02	−0.31	0.757
DI WT/BL	0.16 ± 0.01	0.12 ± 0.01	−1.29	0.198	0.18 ± 0.01	0.12 ± 0.01	−2.25	0.025	0.19 ± 0.01	0.18 ± 0.02	−0.62	0.536
DI WT/IL	0.16 ± 0.01	0.10 ± 0.01	−2.47	0.014	0.17 ± 0.01	0.12 ± 0.01	−2.31	0.022	0.18 ± 0.01	0.18 ± 0.02	0.15	0.883
DI TM/TL	0.05 ± 0.00	0.05 ± 0.01	0.91	0.366	0.06 ± 0.00	0.06 ± 0.01	0.13	0.894	0.08 ± 0.01	0.07 ± 0.01	−1.61	0.109
DI TM/BL	0.06 ± 0.00	0.07 ± 0.01	1.50	0.133	0.07 ± 0.01	0.07 ± 0.01	0.26	0.798	0.09 ± 0.01	0.08 ± 0.01	−1.24	0.215
DI TM/IL	0.06 ± 0.00	0.06 ± 0.01	0.43	0.664	0.06 ± 0.01	0.06 ± 0.01	0.44	0.661	0.09 ± 0.01	0.07 ± 0.01	−1.64	0.102

DI WT/TM: desynchronization (h) between wrist skin temperature and time in movement; DI WT/LE: desynchronization (h) between wrist skin temperature and total light exposure; DI WT/BL: desynchronization (h) between wrist skin temperature and circadian (blue) light exposure; DI WT/IL: desynchronization (h) between wrist skin temperature and infrared exposure; DI TM/TL: desynchronization (h) between time in movement and total light exposure; DI TM/BL: desynchronization (h) between time in movement and circadian (blue) light exposure; DI TM/IL: desynchronization between time in movement and infrared exposure. Statistics: Mann–Whitney U test. Significance level: Pr < 0.002 (Bonferroni correction applied).

**Table 3:** Correlations between markers for sleep and meal times and for meal times.

		Mid-in Bed	Mid-Intake	Mid-in Bed—WTiO	Mid-Intake—WTiO	Breakfast Time	Lunch Time	Dinner Time	Breakfast —WTiO	Lunch —WTiO	Dinner —WTiO
		(GMT0, h)	(GMT0, h)	(GMT0, h)	(GMT0, h)	(GMT0, h)	(GMT0, h)	(GMT0, h)	(GMT0, h)	(GMT0, h)	(GMT0, h)
DI WT-TM	R	−0.073	0.030	0.173	0.200	0.024	−0.019	0.005	0.210	0.188	0.177
	p	0.038	0.410	<0.0001	<0.0001	0.508	0.606	0.898	<0.0001	<0.0001	<0.0001
DI WT-TL	R	−0.062	−0.016	0.168	0.189	0.008	−0.012	−0.057	0.208	0.187	0.158
	p	0.080	0.668	<0.0001	<0.0001	0.827	0.749	0.115	<0.0001	<0.0001	<0.0001
DI WT-BL	R	−0.056	0.008	0.173	0.204	0.044	0.002	−0.045	0.230	0.203	0.168
	p	0.111	0.832	<0.0001	<0.0001	0.221	0.964	0.211	<0.0001	<.0001	<0.0001
DI WT-IL	R	−0.053	−0.017	0.145	0.159	0.016	−0.018	−0.060	0.177	0.158	0.134
	p	0.135	0.648	<0.0001	<0.0001	0.662	0.632	0.104	<0.0001	<0.0001	<0.0001
DI TM-TL	R	−0.010	0.019	0.076	0.066	0.050	0.016	−0.031	0.092	0.083	0.005
	p	0.771	0.605	0.035	0.078	0.166	0.660	0.394	0.001	0.272	0.219
DI TM-BL	R	−0.029	0.039	0.056	0.067	0.060	0.012	0.003	0.088	0.078	0.044
	p	0.402	0.288	0.122	0.072	0.097	0.749	0.938	0.018	0.039	0.236
DI TM-IL	R	0.001	0.038	0.064	0.050	0.067	−0.032	−0.012	0.073	0.037	0.027
	p	0.967	0.303	0.077	0.185	0.066	0.385	0.738	0.049	0.332	0.460

Sleep/food intake marker-WTiO: difference between marker and WTiO, in hours. Spearman’s correlations were considered significant when *p* < 0.007 (Bonferroni correction applied).

## Data Availability

Data supporting reported results can be obtained upon request from the Authors.

## Supplementary Materials

The following supporting information can be downloaded at: https://www.mdpi.com/article/10.3390/biology11081130/s1, Table S1: Percentage of recordings performed during each month, per location.; Table S2: Bed and meal times, and sleep and food intake markers for weekdays and weekends and by location.
