# Supplementary material for: Living at the Wrong Time: Effects of Unmatching Official Time in Portugal and Western Spain
Source: Biology (Basel). 2022 Jul 28;11(8):1130. doi: 10.3390/biology11081130 (PMC9404853; doi:10.3390/biology11081130)
Supplement: Supplementary file 1 [file biology-11-01130-s001.zip › biology-1776079-supplementary.pdf]

Living at the Wrong Time: Effects of Unmatching Official Time in Portugal and Western Spain

María-Ángeles Bonmatí-Carrión [1,2,*], Elvira Casado-Ramirez [2,3], María-Teresa Moreno-Casbas [2,3],
Manuel Campos [2,4], ModulEN Consortium [†], Juan Antonio Madrid [1,2] and Maria-Angeles Rol [1,2,*]

Supplementary information
Materials and Methods

	West Spain	Portugal
**Jan**	16.00	13.33
**Feb**	16.32	11.85
**Mar**	14.63	12.59
**Apr**	13.95	17.04
**May**	13.61	11.85
**Jun**	1.70	3.70
**Jul**	0	0
**Aug**	0.34	0
**Sep**	0	1.48
**Oct**	3.74	11.11
**Nov**	10.88	9.63
**Dec**	8.84	7.41

Table S1. Percentage of recordings performed during each month, per location.

Ambulatory monitoring device

The Kronowise 3.0 device is provided with (i) a temperature sensor with a precision of ± 0.1°C at 25°C and a resolution of 0.0635°C; (ii) a triaxal calibrated MEMS-accelerometer with a linear and equal sensitivity along the three axes, with a range of ± 2 g and a sensitivity of 0.001 g and (iii) three light sensors on the front that determine visible, infrared, and blue radiation, with a range of between 0.01 and 43,000 lux, 16 bits of resolution, an internal auto-setting according to the luminance level, and suppression of flicker at 50/60 Hz. The infrared sensor was sensitive to radiation from 800 to 1,070 nm, whereas the blue light detector was equipped with a Gaussian filter, which eliminates all visible radiation below 440 and over 500 nm. These wavelengths match the sensitivity of melanopsin retinal ganglion cells (460–480 nm) [1] (for further information about the device and motor-related variables, see [2]). The temperature sensor is housed in a separate chamber to avoid thermal interference from the battery and electronic components, with a metal plate in contact with the skin. The triaxial calibrated MEMS-accelerometer with a linear and equal sensitivity along the three axes, with a range of ± 2 g and a sensitivity of 0.001 g. The y axis of the device was aligned with the long axis of the radius; the x-axis corresponds to the radial–ulnar axis and the z-axis to the palmar–dorsal axis. The default sampling frequency was set at 10 Hz. From the accelerometer output, a total of five groups of motor-related variables were recorded:
(a) tilt of the x, y, and z-axis, as the angle between each axis and the horizontal plane, expressed in °, which allows posture changes to be determined during conditions of immobility (i.e., sleep);
(b) the sum of the degrees of change between the current and previous axis position;
(c) the area under the curve, integrating the composite acceleration values per epoch; this variable indicates movement velocity and strength, but not the duration or frequency;

(d) time in movement, as the cumulative time above a very low threshold (0.05 g) in periods of 0.1 s, in which a movement on any of three axes was detected; (e) the area under the curve for individual x, y, and z acceleration, in order to discriminate among types of motor activity (i.e., walking, running, typing, etc.).

Communication with the ACM device was established using Kronoware 10.0 software (Kronohealth SL, Spain) via a USB port. The data were converted into a text file for later analysis using the chronobiological software *Circadianware*, implemented on the *Kronowizard* cloud platform (https://kronowizard.um.es/, University of Murcia).

Circadian parameters

Non-parametric phase markers: L10 or M10 were calculated as daytime phase markers (DPM, the mid-point timing for the 10 consecutive hours with the lowest/highest values, respectively) for those variables with low (WT and sleep probability) or high (MA, TM, TAPL, and light exposures) values during the day, respectively. L5 or M5, as night time phase markers (NPM, the mid-point time of the 5 consecutive hours with the lowest/highest values, respectively) for variables with low (MA, TM, TAPL, and light exposures) or high (WT and sleep probability) values during the night, respectively.
V-day represent the mean value for those 10 consecutive hours with highest (=VM10 for MA, TM, TAPL and light exposure) or lowest (=VL10 for WT and sleep) values. V-night represent the mean value for those 5 consecutive hours with lowest (=VL5 for MA, TM, TAPL and light exposure) or highest (=VM5 for WT and sleep) values. Relative amplitude was normalized after calculated as follows: $RA = \frac{VM10-VL5}{VM10+VL5}$ for MA, TM, TAPL and light exposure and $RA = \frac{VM5-VL10}{VL10+VM5}$.

The interdaily stability (IS) quantifies the invariability between the different days and the intradaily variability (IV) gives an indication of the fragmentation of the rhythm, with the frequency and extent of transitions between different values of the variables (for equations, see [3]. Circadian function index (CFI) was calculated as: $CFI = \frac{IS+IV+RAN}{3}$. From the Fourier analysis, the first and the accumulative power of the first 11 harmonics were obtained and its ratio was calculated in order to obtain the circadianity index (CI).

Reported meal and sleep times

Information about the (translated) questions included regarding sleep and meal times:
What time do you usually get up on weekdays? ____________
What time do you usually go to bed on weekdays? ____________________
What time do you usually get up on public holidays? ___________________
What time do you usually go to bed on public holidays? _____________

**Schedule**	**Week Days**	**Free Days**
**Breakfast Time**		
**Mid-morning Meal Time**		
**Lunch Time**		
**Dinner Time**		

Results

Daily schedule: Bed and meal times

Table S2. Bed and meal times, and sleep and food intake markers for weekdays and weekends and by location.

	Bed and meal times							
	Weekdays				Weekend			
	West Spain	Portugal	**Z**	**Pr**	West Spain	Portugal	**Z**	**Pr**
Bed time (GMT0, h)	22:34 ± 0:04	22:43 ± 0:07	-1.126	0.260	22:43 ± 0.08	22:48 ± 0.08	-0.42	0.675
Get-up time (GMT0, h)	7:12 ± 0:04	7:33 ± 0:06	-2.324	0.020	7:38 ± 0.07	7:52 ± 0.07	-1.32	0.187
Mid 'in bed' (GMT0, h)	2:54 ± 0:03	3:06 ± 0:05	1.56	0.118	3:10 ± 0.06	3:18 ± 0.06	0.82	0.413
Mid 'in bed'-WTiO (h)	7:30 ± 0:09	6:59 ± 0:11	-2.11	0.030	7:47 ± 0.15	7:12 ± 0.15	-2.33	0.020
Breakfast time (GMT0, h)	7:57 ± 0:03	8:06 ± 0:05	-1.289	0.197	8:10 ± 0.06	8:16 ± 0.06	-0.68	0.497
Mid-morning snack time (GMT0, h)	10:40 ± 0:07	10:13 ± 0:04	-2.364	0.018	10:40 ± 0.12	10:13 ± 0.12	-2.49	0.013
Lunch time (GMT0, h)	12:55 ± 0:02	12:19 ± 0:03	-7.6	**<0.001**	13:01 ± 0.04	12:25 ± 0.04	-8.24	**<0.001**
Dinner time (GMT0, h)	19:58 ± 0:03	19:25 ± 0:03	-5.96	**<0.001**	20:00 ± 0.06	19:27 ± 0.06	-5.89	**<0.001**
Mid intake (GMT0, h)	13:58 ± 0:03	13:45 ± 0:03	-2.54	0.011	14:05 ± 0.05	13:51 ± 0.05	-2.66	**0.008**
Mid intake – WTiO (h)	-5:27 ± 0:09	-6:20 ± 0:11	-3.41	**<0.001**	-5:20 ± 0.15	-6:14 ± 0.15	-3.53	**<0.001**
Mid fast (GMT0, h)	1:58 ± 0:03	1:45 ± 0:03	-2.54	0.011	2:05 ± 0.05	1:51 ± 0.05	-2.66	**0.008**
Mid fast – WTiO (h)	6:32 ± 0:09	5:39 ± 0:11	-3.41	**<0.001**	6:39 ± 0.15	5:45 ± 0.15	-3.53	**<0.001**

(Sleep/food intake) marker-WTiO: difference between each phase marker and WTiO, in hours. Negative values indicate that the phase marker occurs earlier than WTiO. Positive values indicate that the phase marker occurs later than WTiO. Greater values indicate later occurrence with respect to WTiO. Statistics: Mann-Whitney U test. Significance level: Pr < 0.008 (Bonferroni corrected).
